# Understanding physical literacy in the context of health: a rapid scoping review

**DOI:** 10.1186/s12889-020-09583-8

**Published:** 2020-10-19

**Authors:** Katie Cornish, Gloria Fox, Trina Fyfe, Erica Koopmans, Anne Pousette, Chelsea A. Pelletier

**Affiliations:** 1 Health Research Institute, University of Northern British Columbia, Prince George, British Columbia Canada; 2 Population and Preventive Public Health, Northern Health, Prince George, British Columbia Canada; 3 Northern Medical Program, University of Northern British Columbia, Prince George, British Columbia Canada; 4 Promotion of Wellness in Northern British Columbia (WINBC), Clinical Faculty, Northern Medical Program, University of British Columbia, Medical Staff, University Hospital of Northern British Columbia, Northern Health, Prince George, British Columbia Canada; 5 School of Health Sciences, University of Northern British Columbia 3333 University Way, Prince George, British Columbia V2N 4Z9 Canada

**Keywords:** Physical activity, Physical education, Fundamental movement skills, Health promotion, Population health, Integrated knowledge translation, Exercise

## Abstract

**Background:**

Physical literacy is a multidimensional concept that describes a holistic foundation for physical activity engagement. Understanding the utilization and effectiveness of physical literacy in the context of health and the health care setting will support clinical and population health programming. The purpose of this rapid scoping review was to: 1) map the conceptualization of physical literacy as it relates to health; 2) identify and describe the utilization of physical literacy in the context of health and engagement of health care providers; and 3) better understand the relationship between physical literacy, physical activity, and health.

**Methods:**

Following established scoping review methods adapted for a rapid review approach, we searched electronic databases Medline OVID, CINAHL Ebsco, PsycInfo Ebsco, Web of Science ISI, and ERIC Ebsco from conception until September 2019. Tabulation coding was used to identify the key themes across included articles and synthesize findings. The review follows an integrated knowledge translation approach based on a partnership between the health system, community organizations, and researchers.

**Results:**

Following removal of duplicates, our search identified 475 articles for title and abstract screening. After full text review, 17 articles were included (12 original research papers and five conceptual or review papers). There was near consensus among included papers with 16 of 17 using the Whiteheadian definition of physical literacy. There was limited involvement of health care providers in the concept of physical literacy. Physical literacy was connected to the following health indicators: BMI and body weight, waist circumference, cardiorespiratory fitness, physical activity, and sedentary behaviour. The primary demographic focus of included studies was children and there was a conceptual focus on the physical domain of physical literacy.

**Conclusions:**

Despite growing popularity, the empirical evidence base linking physical literacy and health outcomes is limited and the relationship remains theoretical. Physical literacy may present a novel and holistic framework for health-enhancing physical activity interventions that consider factors vital to sustained participation in physical activity across the life course. Future work should continue to explore the nature and direction of the relationship between physical activity and physical literacy to identify appropriate focused approaches for health promotion.

## Background

### The concept of physical literacy

Physical literacy is a multifaceted concept comprised of affective (motivation and confidence), physical (physical competence), cognitive (knowledge and understanding), and behavioural (engagement in physical activities for life) domains [1]. These four domains together embody a holistic approach to physical activity that considers the social processes associated with lifelong learning [2, 3].

As a relatively new concept conceived by Whitehead in 1993, measuring and understanding physical literacy is of increasing interest in the fields of education, sport, recreation, and population health [4]. Along with this emergent interest in the concept of physical literacy is ongoing debate and ambiguity regarding its definition. A conceptual analysis conducted by Young and colleagues [3] identified no consistency in how physical literacy is understood or used and the authors suggest the concept exists along a ladder of abstraction. Many models of physical literacy focus on sport development for children and youth, reflecting the uptake of the construct by various sport organizations globally including the Sport for Life Society, Physical Literacy for Life, Sport Australia, Sport England, and the Society of Health and Physical Educators (SHAPE America) in the United States [5–10]. In the literature, physical literacy itself is frequently seen as the outcome or endpoint, with a primary focus on fundamental movement skills in school-age children, rather than as a unique and individual process that evolves across the life course as originally conceived by Whitehead [3, 11, 12].

### Physical literacy, physical activity, and health

Physical literacy is increasingly used as a tool to support population health in Canada with inclusion in ParticipACTION report cards [13] and as one the areas of focus in Canada’s Common Vision for increasing physical activity and decreasing sedentary living [14]. In British Columbia and across Canada, Sport for Life is facilitating the implementation of the Physical Literacy for Communities initiative that aims to advance the development of physical literacy, increase physical activity levels, and promote health and well-being through intersectoral partnership [15]. Although some of the individual tenets of physical literacy have been linked to increased physical activity participation across the life course [16], the concept of physical literacy as a whole is not well studied in the context of health [2]. There is a dominant research focus on the development of measurement tools to assess and deconstruct each of the included physical literacy domains [17, 18]. Although the relationship between physical literacy, physical activity, and health have been demonstrated conceptually [19], it is important to understand the scope and depth of empirical evidence to support evidence-based decision making by the health sector.

Health care providers are key messengers, advocates, and promoters of health. Integrating preventive medicine strategies, such as physical activity promotion, with primary care is challenging [20–22]. Specific emphasis has been placed on exercise prescription and brief counselling interventions by health care providers for clinical populations, such as those utilized in the ‘Exercise is Medicine’ initiative; these interventions demonstrate inconsistent improvements in and maintenance of physical activity levels [23]. A prescriptive approach overlooks social factors influencing physical activity and lifestyle choices and may not consider engagement in physical activity for social and enjoyment outcomes, which are salient facilitators of physical activity engagement [24]. Physical literacy may present a more nuanced, reflexive, and holistic way to incorporate physical activity promotion into the health care setting where context, psychosocial circumstance, individual ability, and knowledge are considered.

Linking multiple settings for promotion of physical activity increases the feasibility and likelihood of changing behaviour [25] and finding strategies to link community and clinical intervention settings [26] may be prudent to ‘move the needle’ on physical inactivity. Thus, if the goal of increasing physical literacy is to develop a healthy population and reduce noncommunicable disease, the health care setting is an important focus for potential intervention. A clear understanding of the construct of physical literacy within the context of health is necessary in order to understand its relevance to the health sector and ultimately support population health.

### Project aims and research questions

This rapid scoping review aims to develop an understanding of physical literacy within the context of health by answering the following research questions:

1. How does current literature define and/or conceptualize physical literacy as it relates to health?
2. In what areas of the health sector is the term ‘physical literacy’ utilized and how are health care providers engaged with this construct?
3. Does existing literature describe a relationship between physical literacy, physical activity levels, and improved health outcomes, and if so, what are the key findings?

## Methods

### Design and project team

A rapid scoping review approach was used to collect and synthesize information describing how physical literacy is defined and utilized in the context of health literature, and to identify physical literacy interventions within the health sector. A rapid review technique was chosen because it allows for rapid collection of evidence to map the field of study to inform the health system and other stakeholders to support decision making [27–29]. This review was conducted to support Health Milestones in Physical Literacy for Communities initiatives (see: https://physicalliteracy.ca/physical-literacy-for-communities-british-columbia-initiative/) and to inform implementation approaches for a regional physical activity strategy, aligned with provincial and federal objectives. Our scoping review follows the rigorous methodology established by Arksey and O’Malley [29] and further developed by Levac, Colquhoun, and O’Brien [30] with modifications for timeliness (rapid review) detailed below. A protocol was developed using the Preferred Reporting Items for Systematic Reviews and Meta-Analysis Protocols (PRISMA-P; Additional file 1) [31]. The project was originally designed to be completed in 3 months, but faced delays due to factors outside of the control of our research team (e.g., faculty strike, COVID-19 pandemic). We followed an integrated knowledge translation approach, which involves the collaborative and equitable partnership between researchers and knowledge users throughout the research process [32]. Accordingly, our team includes researchers and research trainees (CP, KC), a health sciences librarian (TF), knowledge synthesis expertise (EK, TF), non-profit organization (AP), clinician (AP), and health system decision maker in population and preventive public health (GF). This manuscript has been prepared adhering to the PRISMA extension for scoping reviews (PRISMA-ScR; Additional file 2) [28].

### Search strategy and screening

The search strategy was developed by a health sciences librarian (TF) using key words and subject headings for physical literacy (“physical literacy”) and health (“health, perceived health, public health, population health, physical fitness, wellbeing, and wellness”; Additional file 3). Databases searched included Medline OVID, CINAHL Ebsco, PsycInfo Ebsco, Web of Science ISI, and ERIC Ebsco from conception until September 3, 2019. The reference list of relevant review papers and included articles were hand searched for additional articles. Inclusion and exclusion criteria are presented in Table 1.

**Table 1:** Inclusion and exclusion criteria

Inclusion	Exclusion
(1) Articles that conceptualize or define physical literacy and are reported from a health context will be included. Studies must: a. Have some mention of physical literacy or a physical literacy lens; b. Be reported from or in a health context including tools used in the past in a health setting or applied in the health sector. (2) All peer reviewed academic publications that are conceptual, theoretical, reviews, or original research articles will be included.	(1) Studies that do not conceptualize or define physical literacy; (2) Case reports, conference abstracts, editorial and opinion pieces, book chapters, book reviews, and book synopses; (3) Non-English studies; (4) Articles where full text is not available through University of Northern British Columbia Library or Interlibrary Loans.

Titles and abstracts of identified articles were uploaded to DistillerSR (Evidence Partners, Ottawa) for screening based on inclusion and exclusion criteria. In line with a rapid review approach, items were screened by one reviewer (KC) in two stages: title and abstract, and full text [33–35]. The entire team met to discuss included articles after the first stage of screening (title and abstract) and again to confirm the final list of included papers. During screening, any articles the primary reviewer was unsure about were discussed with a second author (EK) and the principal investigator (CP) until consensus was reached. Included papers were separated into conceptual papers describing the theoretical link between physical literacy and health and primary research articles that measured aspects of physical literacy and health. Consistent with established scoping review methods, a methodological quality assessment of included articles was not conducted [29, 36]. The focus of this review was to map both conceptual articles and original research reports exploring the link between physical literacy and health, thus we felt it would be inappropriate to exclude or rank papers based on study quality.

### Data extraction

Data extraction was performed using a standardized data extraction form in DistillerSR, iteratively revised after piloting and discussion with the research team. Data extraction for conceptual articles included study and author information, design of review or model development, definition or conceptualization of physical literacy used, and the nature of the relationship between physical literacy, physical activity levels, and improved health outcomes. The data extraction form for original research articles included additional extraction points including: the study setting and context, population and sample size, purpose and aim of study, definition or conceptualization of physical literacy, engagement of health care providers, the nature or characteristics of the intervention (where applicable), and key findings or study outcomes.

### Synthesis of findings

Excel tables were used to organize the data and descriptive statistics were used to summarize the number of publications per year, country of origin, and study design [37]. Tables were used to organize extracted information pertaining to the definitions of physical literacy, participant demographic information (original research articles only), health care provider engagement, and key findings. Inductive reasoning and thematic analysis was used to synthesize key themes from the data in response to the specific research questions [38, 39].

## Results

### Literature search and study characteristics

**Fig. 1:**
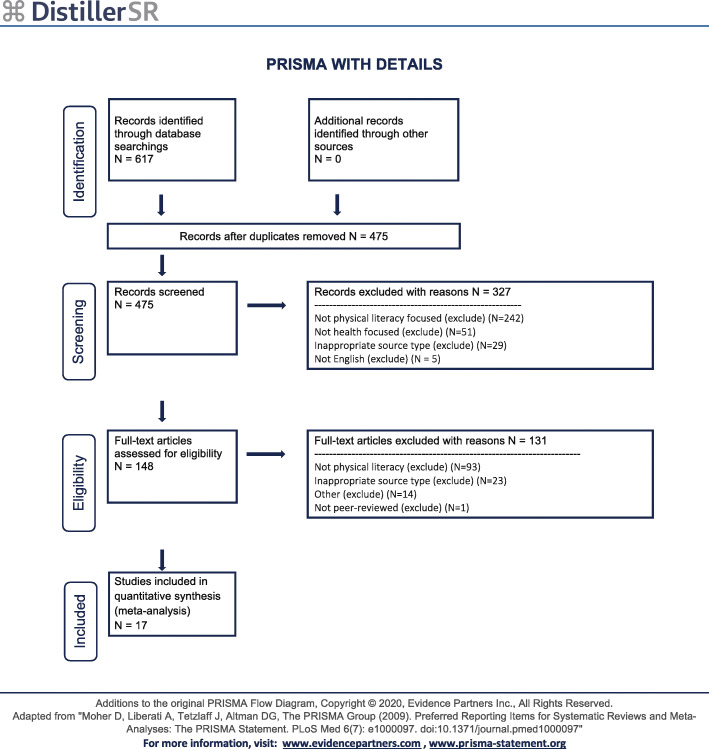
PRISMA flow diagram with details

The electronic database search yielded 617 items total and 475 remained following removal of 142 duplicates. After title and abstract screening, the full texts of 64 articles were reviewed. Seventeen articles were included in the final sample. Twelve included articles were original primary research articles (summarized in Table 2) and five were conceptual (summarized in Table 3). The screening process is depicted in Fig. 1. The years of publication for the total sample ranged from 2015 to 2019, with the majority of articles published in 2018 (*n* = 7). In 2019, there were five articles published. The majority of the research was published and conducted in Canada (*n* = 11, 64%). Two articles were published in the United Kingdom, and one article each was published from Australia, China, Germany, and Austria [41, 44, 45, 52, 54, 55].

**Table 2:** Detailed summary of original research articles (*n* = 12)

Author	Country of origin	Study design	Definition and citations	Aim or purpose	Sample	Health care providers engaged?	Intervention and characteristics	Key findings	Conclusions
Belanger et al. 2018 [40]	Canada	Quantitative – cross-sectional	Whiteheadian	To examine associations between physical literacy (PL) domain scores among children who meet or do not meet Canadian physical activity guidelines (PAG) or sedentary behaviour guidelines (SBG).	Children aged 8–12 (*n* = 2956)	No	CAPL scores were compared between children who did and did not meet the daily recommended PAG.	Only 20% of sample met PAG. Children meeting PAG had higher PL domain scores for physical competence and motivation and confidence. Children were more likely to meet PAG if they met the minimum recommended level of the physical competence and motivation and confidence domains. Boys had significantly higher PL scores overall, With the exception of the knowledge & understanding domain. Boys were more likely to meet PAG than girls.	Children were more likely to meet PAG and SBG if they achieved the minimum recommended level of PL domain scores, specifically in the physical competence and motivation and confidence domains. Findings suggest associations between PL and the degree to which children adhere to PAG and SBG.
Choi et al. 2018 [41]	Other (China)	Quantitative – cross-sectional	Whiteheadian	To examine the relationship between perceived PL and physical activity (PA) levels in adolescents studying in various secondary schools in Hong Kong.	Adolescent secondary school students aged 12–18 (*n* = 1945)	No	Questionnaires measured perceived PL and time spent physically active in past 7 days. Questions assessed walking, school activities, house work, and active transport. Attributes measured: sense of self and self-confidence, self-expression and communication with others, and knowledge and understanding. Demographic info and socio-economic status (SES) collected.	All attributes (sense of self and self-confidence, self-expression and communication with others, and knowledge and understanding) of perceived PL were found to be significant predictors for recreational PA levels. SES was divided into three levels, with middle income group having highest correlation between perceived PL and PA levels.	Study concludes relationship exists between perceived PL and PA levels, and individual factors (age, gender, and SES) impact this relationship. Authors argue the concept of PL should be introduced to adolescents by assigning them the responsibility of designing their chosen PA and discussing the importance of PA for lifelong health.
Comeau et al. 2017 [42]	Canada	Quantitative– cross-sectional	Fundamental movement skills	To evaluate the association between fundamental movement skills (FMS) and health indicators using the PLAYbasic and Passport for Life tools and to evaluate whether the associations between FMS and health indicators were different between the two tools.	Children aged 9–12 years old (grades 4–6) (*n* = 145)	No	FMS were evaluated using the Passport for Life and the PLAYbasic tools. Health metrics (BMI, waist circumference, weight, grip strength, and cardiorespiratory fitness) were compared between each test.	FMS is significantly and independently associated with health metrics regardless of age or sex (BMI, waist circumference, weight, grip strength, and cardiorespiratory fitness). The results when comparing the two FMS measurement tools showed no difference.	Children with a high level of FMS display a better health indicators profile when compared to children with poor FMS. Tools used to measure FMS (PLAYbasic or Passport for Life) do not impact the associations observed.
Delisle Nyström et al. 2018 [43]	Canada	Quantitative – cross-sectional	Whiteheadian	To determine the associations among the four domains of PL stratified by weight status.	Children aged 8 to 12y (*n* = 8343)	No	BMI/waist circumference measured. Four domains measured along with overall PL using cardiorespiratory fitness (PACER), muscular strength (handgrip, abdominal plank test) and the sit-and-reach test (flexibility). Daily behaviour measured using pedometer and questionnaire items. Motivation and confidence and knowledge and understanding measured using a questionnaire.	In the healthy-weight group, positive associations were found among all PL domains. Results suggest that motivation and confidence are important correlates of modified physical competence and daily behaviour, irrespective of weight status. Healthy-weight children scored higher in all four domains and overall PL in comparison to overweight/obese children, although the differences were small to negligible.	Findings align with current research and future interventions aimed at improving PL do not need to be tailored based on weight status, although longitudinal studies are needed to confirm these conclusions.
Gibson et al. 2019 [44]	United Kingdom	Qualitative – formative concept testing	Whiteheadian	The purpose of the research is to explore how health and well-being specialists engage with the concept of ‘food and physical literacy’ (FPL).	Professionals from teaching, community development, public health, health and well-being, mental health, social support, active travel, and recreation. (*n* = 36)	Assessed how professionals engage with the concept of FPL.	Participants attended a workshop on FPL and participated in a focus group discussion on the following themes: professionals’ initial opinions on the concept, its applicability to their practice, and the perceived barriers and facilitators to the advancement of the concept. A questionnaire followed to give participants the opportunity to provide feedback.	Participants’ reactions to the concept were mixed and identified possible barriers, including the name of the concept, its narrow scope, and the perceived intangibility as a substantial number of participants [12] struggled to see the practical application. Despite the barriers, some participants suggested that they were receptive to new concepts, and favoured the inclusive nature of FPL.	FPL is proposed as providing a novel and sensitive resource for thought and for future action. The authors recommend more research on the developing concept of FPL.
Holler et al. 2019 [45]	Other (Austria)	Quantitative - non-randomized control trial	Whiteheadian	To assess the effects of a holistic physical exercise training intervention on PL in physically inactive adults and to identify sociodemographic parameters affecting changes in PL.	Physically inactive adults (*n* = 31) intervention group, (*n* = 30) control.	Physicians assisted with recruitment of participants using motivational interviewing.	Intervention group (IG) participated in a holistic physical exercise training intervention once weekly for 15 weeks, while matched control group (CG) did not. BMI collected at start and end of study. PL was evaluated by a questionnaire assessing PA behaviour, attitude towards a physically active lifestyle, exercise motivation as well as exercise knowledge and exercise self-confidence/self-efficacy.	Findings indicate participation in a holistic exercise training program increases total PL score and selective PL domains (physical activity behaviour and exercise self-confidence/self-efficacy) in physically inactive adults. Improvements in PL are positively correlated with BMI values at baseline. No changes were found concerning attitude towards a physically active lifestyle, exercise knowledge or motivation.	Validated measurement tools are not available for measuring adult PL and therefore this should be taken into account when interpreting these findings. Further research is necessary to determine the psychometric properties for this PL questionnaire.
Kwan et al. 2019 [46]	Canada	Quantitative - quasi-experimental design	Whiteheadian	The purpose of this study was to evaluate the effectiveness of a pilot PL based intervention in emerging adults.	First-year university students (17+) transitioning directly from high school to living in residence. IG (*n* = 26), HAL-CG (*n* = 20) and CG (*n* = 23).	No	PL domains were measured for all participants at baseline and follow-up. The IG took part in a 12-week program (PLUS intervention) designed to facilitate novel movement skills in a fun and engaging group-based environment. Measures included: PLAYfun tool for movement competence and confidence, motivation, and knowledge and understanding were measured via questionnaire.	PL based interventions can be effective in enhancing overall PL in the emerging adult population. Findings suggest that there was no overall change with respect to movement competence, small-to-moderate effect sizes were evident in the time by condition interactions for motivation, confidence, and knowledge and understanding. While the intervention was successful in maintaining and improving the psychological domains of PL, there were no observable changes in motor competence.	Given the typical decline in PA with age, the PLUS intervention may be a promising approach in promoting the maintenance of lifelong engagement in PA.
Lang et al. 2018 [47]	Canada	Quantitative – cross-sectional	Whiteheadian	The aim of this study was to assess the relationships between cardiorespiratory fitness and components of PL among Canadian children aged 8–12 years.	Children aged 8–12 years (*n* = 9393)	No	PL was measured using the CAPL, CSAPPA, and 20 m shuttle run. Component measures of each domain: physical competence (total domain score, handgrip, plank, sit-and-reach, BMI, wait circumference, CAMSA), daily behaviour (total domain score, avg. daily steps, self-reported screen time, avg. days/week meeting guidelines), motivation and confidence (total domain score, benefits and barriers, activity level compared to peers, skill level compared to peers, CSAPPA adequacy and predilection scores), knowledge and understanding (total domain score, CAPL questionnaire score).	Cardiorespiratory fitness (CRF) is strongly associated with all components of PL in school-aged Canadian children. Participants in high CRF groups demonstrated better scores across all domains of PL in comparison with peers in lower CRF groups, regardless of age and gender. The strongest associations were identified between CRF and physical competence, followed by motivation and confidence, daily behaviour, and knowledge and understanding.	Preliminary evidence supports CRF as a predictor of PL outcomes. Future studies should aim to replicate these results in different populations, and to identify the sensitivity and specificity of using CRF to screen for children with low PL levels.
MacDonald et al. 2018 [48]	Canada	Quantitative – cross-sectional	Whiteheadian	The purpose of this study was to investigate the association of age, gender, and physical competence components of children’s PL levels, with perceived adequacy in and predilection for physical activity	Children aged 8–12 years (*n* = 8530)	Authors make recommendations for health care providers by suggesting physical fitness should remain the focus of practitioners targeting physical activity promotion in youth.	Measures of PL included: Progressive Aerobic Cardiovascular Endurance Run (PACER), Canadian Agility and Movement Skill Assessment (CAMSA), sit and reach, handgrip, plank, and body mass index) and children’s perceived adequacy and predilection toward physical activity as measured by subscales from the Children’s Self-Perceptions of Adequacy in and Predilection for Physical Activity scale (CSAPPA).	Results demonstrated CRF, as measured by the PACER, was moderately related to children’s perceived levels of adequacy and predilection. Findings align with literature stating both boys and girls tend to prefer non-physical activities as they age.	It is unclear whether high levels of physical competence in PL lead to higher affective states or vice versa. The findings support the assertion that effective teachers and coaches will consider both the physiological and psychological makeup of a child to promote optimal PA participation.
Millington 2015 [49]	Canada	Qualitative – multiple methods approach	Whiteheadian	This study aims to understand how video games such as Wii Bowling are being used in retirement centres and the implications of fusing technology and PL .	Four retirement centres offering Nintendo Wii as part of their activity programming. Staff (*n* = 10) and residents (*n* = 8)	No	Methods used in this research included semi-structured interviews with retirement centre staff (*n* = 10), semi-structured interviews with older persons residing at retirement centres (*n* = 8), and observation of exergaming in action.	Interactive games such as Wii Bowling are perceived by staff and residents as useful in the pursuit of active aging. Some games present challenges for older persons, both in their physical demands and in the need for gamers to successfully blend media and physical literacies. Residents noted sore muscles, aches, and arthritis flare-ups. Technologies presented challenges for staff as they struggled to blend media and physical literacies.	Exergaming may prove to be a useful tool in promoting PL and active aging in older adults.
Pohl et al. 2019 [50]	Canada	Quantitative – cross-sectional	Whiteheadian	The purpose of this study is to compare the PL of children with epilepsy with that of a reference population of Canadian children without known health conditions.	Epileptic children aged 8–12 years with at least one seizure in the last 12 months. IG (*n* = 35), and CG (*n* = 228)	Neurology clinic was location for recruitment	CAPL assessment was conducted (daily behaviour monitored with a pedometer), and self-reported PA and screen time was recorded in IG and CG.	Only 11% of children with epilepsy met recommended PL scores, significantly fewer than the control group. Daily behaviour was not significantly different, but epileptic children had significantly lower agility and movement skills. Epileptic children matched their peers in the knowledge and understanding domain, yet scored higher than their peers in the motivation and confidence domain.	Children with epilepsy had significantly lower PL levels than their peers without known health conditions. Increased PA in children with epilepsy may improve their general long-term health, decrease anxiety and depression, and improve self-esteem and social integration while boosting neurocognitive skills.
Saunders et al. 2018 [51]	Canada	Quantitative – cross-sectional	Whiteheadian	The purpose of this study was to identify the domains of PL associated with key modes of sedentary behaviour (SB) among Canadian children.	Children aged 8–12 years (*n* = 8307)	No	PL was assessed using the CAPL. SB was broken into screen-based s, non-screen, and total SB. All were assessed via self-report questionnaire.	SB is associated with total PL, as well as motivation and confidence, knowledge and understanding, and physical competence domains. Motivation and confidence demonstrated the strongest association with screen-based modes of SB, while knowledge and understanding showed positive associations with non-screen SB, and negative associations with screen-based SB. Girls were more likely to meet screen-time guidelines than boys.	Interventions should be tailored to participant gender and age, which are associated with multiple modes of SB in this age group.

*Abbreviations*: *CAPL* Canadian assessment of physical literacy, *CG* Control group, *CRF* Cardiorespiratory fitness, *CSAPPA* Children’s Self-Perceptions of Adequacy in and Predilection for Physical Activity, *FMS* Fundamental movement skills, *FPL* Food and physical literacy, *IG* Intervention group, *PA* Physical activity, *PAG* Physical activity guidelines, *PL* Physical literacy, *SB* Sedentary behaviour, *SBG* Sedentary behaviour guidelines

**Table 3:** Detailed summary of conceptual or review articles (*n* = 5)

Author	Country of origin	Study design	Definition	Aim or purpose	Health care providers engaged?	Key findings	Conclusions
Cairney et al. 2019 [19]	Canada	Literature review	Whiteheadian	The purpose of this work is to present a conceptual model positioning physical literacy (PL) as a health determinant, and present evidence in support of PL as a health determinant, drawing on research largely from outside physical education.	No	PL is a gateway to increasing physical activity (PA), which means PL must also be a necessary determinant of health via its impact on PA. Authors are developing a conceptual model positioning PL as a health (and disease) determinant, based on how professional communities (public health) might think about PL in the context of health promotion and disease prevention. This connection lacks direct empirical connectivity between PL and health outcomes, but authors feel there is sufficient evidence to warrant further research into the relationship.	By providing an evidence-informed model, it will encourage further discussion and stimulate empirical research on PL and the relationship to health.
Demetriou et al. 2015 [52]	Germany	Systematic Review	Whiteheadian	This review provides detailed information on the aims, the theoretical background, content, design, methodological quality, and effectiveness of school-based interventions aiming to influence students’ health-related fitness knowledge	No	The primary aim of intervention programmes was the prevention of chronic heart disease risk factors. This overall aim was split up in more specific targets: increase PA and fitness, reduction of risk factors for obesity, nutrition behaviour, and psychological variables such as motivation and attitudes towards PA. School-based PA interventions can change students’ health-related fitness knowledge levels. Intervention programmes addressing adolescents were more frequently able to change their health-related fitness knowledge in comparison to studies that aimed to enhance children’s health-related fitness knowledge levels.	Authors recommend a standardised, validated and reliable measurement instrument to assess students’ health-related fitness knowledge is needed in order to compare the studies’ effects and also changes between cohorts of students
Dudley et al. 2017 [2]	Canada	Literature Review	Whiteheadian	To present a new model of PL policy considerations for decision makers in public health, recreation, sport, and education. Definitions of PL and the wider construct of literacy were reviewed in order to establish common pillars of PL in an applicable policy model.	No	Authors state there are problems with connecting fundamental movement skills (FMS) to PL in that some practitioners, and by default, policymakers think FMS can be taught in isolation. Most attempts to rationalize these skills into public health, sport, or education resources have met controversy in the PL context because they fail to capture the broader PL components of moving for play, enjoyment, or recreation.	PL is still far from having the empirical weight to be substantiated as best practice in reduction of non-communicable disease or the promotion of PA. Future research should focus on clearly articulating the definitions, philosophical assumptions, and expected outcomes prior to evaluating the effectiveness of this emerging concept.
Edwards et al. 2017 [53]	United Kingdom	Systematic review	Whiteheadian	The purpose of this systematic review was to conduct a systematic review of the PL construct as reflected in the contemporary research literature	No	Few of the proposed relationships and causal associations claimed for PL have been empirically tested to date, although such trials are currently underway. This paper is the first to provide a review of the core attributes of the PL construct, including the defining properties of PL, the philosophical foundations, and the theoretical associations of the construct. Seventy percent of articles that referred to the term PL adopted a Whiteheadian perspective.	Researchers need to operationalize PL and generate meaningful, measurable differences that will eventually be the arbiter of what PL is and how it works.
Fortnum et al. 2018 [54]	Australia	Scoping review	Whiteheadian	To review the evidence relating to the individual components of PL (4 domains) in children with behavioural and emotional mental health disorders (MHDs). *n* = 68 studies included.	Physiotherapists should be included in the management of children with MHDs to increase the likelihood of children becoming physically literate and to help moderate the negative impacts of MHDs on physiological health.	Research is predominantly based around the physical competence domain, with 62 articles exploring the topic; other domains are not as well represented in the literature. Daily behaviour was assessed in 12 studies, motivation and confidence in 2 studies, and knowledge and understanding in 1 study. Children with MHDs appear to have lower PL levels than children without MHDs and can be observed across the daily behaviour domain, as well as the motor proficiency, aerobic fitness, muscular strength and muscular endurance components.	Children with MHDs are more likely to exhibit lower levels of PL than children without a MHD diagnosis, as such it is likely children with MHDs require additional support and tailored interventions to promote PL. A comprehensive PL assessment should be included in standard care for clinicians working with children with MHDs.

*Abbreviations*: *FMS* Fundamental movement skills, *MHD* Mental health disorders, *PA* Physical activity, *PL* Physical literacy

The majority of primary research articles (*n* = 10) used quantitative methodologies and two used qualitative approaches. Study designs were cross-sectional (*n* = 8; [40–43, 47, 48, 50, 51]), quasi-experimental (*n* = 1; [46]), a multi-methods observational approach (*n* = 1; [49]), a non-randomized control trial (*n* = 1; [45]), and a formative conceptual evaluation (*n* = 1; [44]). The five conceptual articles included two narrative review articles [2, 19], and three systematic or scoping reviews [52, 54, 55]. A total of nine articles explored physical literacy in children, 8–12 years [40, 42, 43, 47, 48, 50, 51], and adolescents, 12–18 years [41, 46]. One study explored physical literacy in inactive adults [45], and one explored physical literacy among older adults living in long term care [49].

### Definition of physical literacy

Sixteen of 17 included articles (94%) used the Whiteheadian definition of physical literacy: “physical literacy is defined as the motivation, confidence, physical competence, knowledge and understanding to value and take responsibility for engagement in physical activities for life” (p.11) [12]. This is the same definition used by the International Physical Literacy Association [56] and the two are often used interchangeably due to their similarities. While the remaining article offered no definition of physical literacy, it applied a physical literacy lens and instead opted to use the term ‘fundamental movement skills’ [42].

Seven included articles focused on the physical domain of physical literacy [40, 42, 43, 47, 48, 50, 51]. The affective, cognitive, and behavioural domains were typically studied via questionnaire, with few items addressing each domain. In five studies, questionnaire results for the behavioural domain were supplemented with 7 days of pedometer data [40, 43, 47, 50, 51]. None of the articles focused on the affective, cognitive, or behavioural domains, and two studies did not report on these domains at all [42, 46].

Physical literacy was most commonly measured with a standardized questionnaire such as the Canadian Assessment of Physical Literacy (CAPL; *n* = 6) or PLAYbasic/PLAYfun (*n* = 2). Table 4 summarizes the included studies based on physical literacy domain studied and measures used.

**Table 4:** Measurement summary by domain of original research articles (*n* = 12)

Authors	Physical competence (physical)	Motivation and confidence (affective)	Knowledge and understanding (cognitive)	Engagement in physical activity for life (behavioural)
Belanger et al. 2018 [40]	CAPL, CAMSA	CAPL	CAPL	CAPL (pedometer step counts 7 days; self-reported activity)
Choi et al. 2018 [41]	IPAQ-A	PPLI	PPLI	PPLI
Comeau et al. 2017 [42]	Passport for Life and PLAYbasic	Not reported	Not reported	Not reported
Delisle Nyström et al. 2018 [43]	CAPL, CAMSA	CAPL	CAPL	CAPL (pedometer step counts 7 days; self-reported activity)
Gibson et al. 2019 [44]	Not reported	Not reported	Not reported	Not reported
Holler et al. 2019 [45]	Questionnaire informed by ACSM/AHA, SMS28, SIMS, FKB-20, BREQ-2, IPAQ-SF, Stanford Five City Study.	Questionnaire informed by ACSM/AHA, SMS28, SIMS, FKB-20, BREQ-2, IPAQ-SF, Stanford Five City Study.	Questionnaire informed by ACSM/AHA, SMS28, SIMS, FKB-20, BREQ-2, IPAQ-SF, Stanford Five City Study.	Questionnaire informed by ACSM/AHA, SMS28, SIMS, FKB-20, BREQ-2, IPAQ-SF, Stanford Five City Study.
Kwan et al. 2019 [46]	PLAYfun tool	BREQ-3, and two items based on recommendations from Bandura (1997, 2006).	BREQ-3	Not reported
Lang et al. 2018 [47]	CAPL, CAMSA	CAPL, CSAPPA	CAPL	SC-StepRx pedometer for 7 days
MacDonald et al. 2018 [48]	CAPL, CAMSA	CAPL, CSAPPA	CAPL	CAPL
Millington 2015 [49]	Semi-structured interviews with retirement centre staff (*n* = 10), and older persons residing at retirement centres (*n* = 8). Observations of exergaming.	Semi-structured interviews with retirement centre staff (*n* = 10), and older persons residing at retirement centres (*n* = 8). Observations of exergaming.	Semi-structured interviews with retirement centre staff (*n* = 10), and older persons residing at retirement centres (*n* = 8). Observations of exergaming.	Semi-structured interviews with retirement centre staff (*n* = 10), and older persons residing at retirement centres (*n* = 8). Observations of exergaming.
Pohl et al. 2019 [50]	CAPL, CAMSA	CAPL	CAPL	CAPL (pedometer step counts 7 days; self-reported activity)
Saunders et al. 2018 [51]	CAPL, CAMSA	CAPL	CAPL	CAPL (pedometer step counts 7 days; self-reported activity)

*Abbreviations*: *ACSM/AHA* American College of Sport Medicine’s/American Heart Association, *BREQ-2* Behavioral Regulation in Exercise Questionnaire-2, *BREQ-3* Behavioral Regulation in Exercise Questionnaire-3, *CAMSA* Canadian Agility and Movement Skill Assessment, *CAPL* Canadian Assessment of Physical Literacy, *CSAPPA* Children’s Self-Perception and Adequacy in and Predilection for Physical Activity, *FKB-20* Body Image Questionnaire, *IPAQ-A* International Physical Activity Questionnaire for Adolescents, *IPAQ-SF* International Physical Activity Questionnaire – Short Form, *PPLI* Perceived Physical Literacy Instrument, *SIMS* Situational Motivation Scale, *SMS28* Sport Motivation Scale

### Physical literacy in the health care setting

Five included articles discussed the engagement or potential engagement of health care providers with physical literacy. Of these five, one article [48] recommended health care professionals should consider children’s physiological and psychological make up to best enhance children’s adequacy and predilection for physical activity. One article [54] suggested physiotherapists should be included in the management of children with mental health disorders to increase the likelihood of them becoming physically literate. Two articles [45, 50] reported physicians assisting in the participant recruitment process, and one study [44] explored how health care providers perceived the concept of food and physical literacy, finding providers found its perceived intangibility, unnecessarily complicated title, and its narrow scope to be conceptual limitations.

### Relationship between physical literacy and health

Seven included primary research studies explored the association between various health indicators and physical literacy [40, 42, 43, 45, 47, 48, 51]. Health indicators including BMI, waist circumference, body weight, grip strength, and cardiorespiratory fitness were found to be predictors of physical literacy levels, specifically within the physical domain [42, 43]. Belanger et al. [40] found children with higher physical literacy scores are more likely to meet daily physical activity and sedentary behaviour recommendations; similarly, physical and affective domains of physical literacy were negatively associated with screen and non-screen sedentary time [51]. Across three primary research articles included in this review, participants considered to be of a healthy weight recorded higher physical literacy scores than those classified as overweight or obese [42, 43, 45]. Participants with higher BMI, and greater waist circumference and weight were more likely to have a lower overall physical literacy score than participants considered to be within the healthy BMI, weight, and waist circumference for their age and height [42, 43]. Findings from one study indicate that children with higher cardiorespiratory fitness scores had greater physical literacy across all domains of physical literacy, regardless of age or gender [47]; another found cardiorespiratory fitness to be linked to children’s perceived levels of adequacy and predilection for physical activity [48].

Two included studies used physical literacy domains as a guiding model for a physical activity intervention. Results from a study conducted by Holler et al. [45] demonstrated improvements in behavioural and affective (self-confidence and self-efficacy) aspects of physical literacy in inactive adults based on a 15-week holistic exercise program. This study worked with physicians to refer their patients into the program using motivational interviewing [45]. Similarly, Kwan et al. [46] conducted a 12-week intervention delivered to first-year university students focused on novel movement skills utilizing group-based environments. Findings indicate a significant increase in overall physical literacy and improvements in domains of motivation and knowledge/understanding in the intervention group when compared to control. This study did not include measures of physical activity participation or health indicators [46].

Two included articles explored physical literacy in children with chronic disease. In a scoping review, Fortnum, Furzer, Reid, Jackson, and Elliott [54] found children with mental health disorders scored lower across the physical and behavioural domains of physical literacy; however, the authors noted insufficient literature to draw conclusions regarding the cognitive and affective domains. Pohl, Alpous, Hammer, and Longmuir [50] measured physical literacy using CAPL and found children with epilepsy scored lower in the physical domain and higher in the affective domains when compared to their peers without epilepsy.

## Discussion

The purpose of this rapid scoping review was to expand our understanding of and map current literature exploring physical literacy within the context of health. Specific objectives were to: 1) determine the definition and conceptualization of physical literacy as it relates to health in the current literature; 2) identify if health care providers are engaged with the construct of physical literacy and in what types of health care settings physical literacy is used; 3) describe the relationship between physical literacy, physical activity, and health. Synthesis of 17 included articles suggest consensus for the definition of physical literacy, a predominant focus on the physical domain of physical literacy, and little meaningful engagement of health care providers in the construct of physical literacy to promote health.

### Concept and measurement of physical literacy

With only one exception, there was consistent use of the Whiteheadian or International Physical Literacy Association definition of physical literacy among included articles. The remaining article used the term ‘fundamental movement skills’ and provided no definition of physical literacy [42]. The findings from this review align with the findings of recent systematic reviews on physical literacy where the vast majority of included articles used the Whiteheadian definition [53, 55]. This indicates the field of study as a whole is moving towards consensus on the definition of physical literacy.

Physical literacy is a cluster of domains (affective, physical, cognitive, behavioural) conceptually linked together. As individual constructs, they are commonly separated or prioritized depending on context or target population. Although this separation may help with measurement from a research and evaluation perspective, it may diminish the holistic intent and philosophical underpinnings of the concept as originally envisioned [3]. The dominant focus on the physical domain in the academic literature suggests a privileged and ableist focus on an individual’s physical prowess, rather than the holism described by Whitehead [3, 11]. This focus may reflect the uptake of physical literacy by sport-focused organizations and relative lack of utilization by health, healthy living, community and social development, or inclusivity (disability) focused organizations. Current measurement tools, including CAPL and PLAYbasic [57, 58] are typically conducted using a survey and by measuring specific fundamental movement skills, and are limited in their measurement of the affective or cognitive domains of physical literacy [59]. For example, the CAPL-2 is made up of three physical competence protocols including plank, Progressive Aerobic Cardiovascular Endurance Run (PACER), and the Canadian Agility and Movement Skills Assessment (CAMSA), and two measures of daily behaviour: pedometer steps for seven consecutive days, and a self-reported questionnaire [57]. A single 22-item questionnaire is then administered to assess the knowledge and understanding, and motivation and confidence domains [57, 60, 61]. While the CAPL-2 is considered a comprehensive assessment, we agree with the critical analysis conducted by Robinson & Randall (2017) in that this measure is largely skill and fitness based and has limited fidelity to physical literacy as envisioned by Whitehead [59].

### Physical literacy, physical activity, and health

Original research studies and systematic reviews within the sample identified positive changes to physical literacy can be made using interventions that target all four domains of physical literacy in children, youth, and adults [45, 46, 52]. The two included intervention studies demonstrated increases in physical literacy with interventions focused on holistic group-based exercise classes [45] and novel movement experiences [46], however there was no direct link with health outcomes reported. While the connection between physical activity and health is clear [62], the link between physical literacy and health is not yet well established or demonstrated in prospective studies. It is unknown if physical literacy leads to better health outcomes, or if people who are healthier have higher levels of physical literacy. Similarly, evidence identified in this review indicates a relationship between physical literacy and physical activity although we are unable to identify the causative factor, for example, which should be the focus of initial intervention – physical activity or physical literacy? Physical literacy is viewed as a concept that creates a condition to engage in physical activity, but it is likely a bidirectional relationship exists in that increasing physical activity will increase physical literacy and vice versa.

The development of physical literacy is based on engagement in activities in a variety of environments and the fundamental need to participate in movement experiences that are safe, nurturing, rewarding, and enjoyable to promote confidence and motivation [4]. Among the included intervention studies, the focus on enjoyment of activity and providing novel movement experiences were essential components contributing to program adherence via group-based activities [46]. Particularly for older adults, the social elements of physical activity, enjoyment, and confidence are important facilitators of physical activity [63–65]. From a health and life course perspective, the current literature focus on physical domains of physical activity may be missing the true strength and potential of the concept of physical literacy to support sustained physical activity engagement.

### Physical literacy in the health care setting

Findings from this review suggest health care providers are not engaged with the construct of physical literacy. The absence of literature examining how health care providers are engaging with physical literacy in practice may indicate the area is understudied, or articles are not being formally published in the academic literature. Nonetheless, this work indicates a gap in the literature examining the application and utilization of the concept of physical literacy in health care practice. If we are to collectively advance the construct and usefulness of physical literacy in health care settings, then we need to understand how health care providers engage with the concept. The healthcare setting is an important environment for the promotion of physical activity, with strategies that include referrals, exercise prescriptions, and brief counselling interventions [66]. Most primary care physicians report asking patients about physical activity (85%), although a lack of knowledge, resources and context-specific tools are barriers to providing more detailed written prescriptions [67]. Physical activity counselling and prescriptions delivered in the health care setting are more effective if they include an assessment of individual needs, motivation and preferences, if social supports are available, and valid behavioural change models are used [68]. Using physical literacy as a concept or framework to guide interventions delivered by or including health care providers would consider the holistic factors impacting physical activity behaviour and well-designed tools or resources could address some of the barriers identified by primary care providers and end-users. Incorporating factors beyond a prescription to be active based on clinical exercise guidelines (time, frequency, intensity, and type) with inclusion of other elements or domains such as enjoyment, motivation, confidence, and knowledge may have greater relevance for populations at increased risk for inactivity including people in rural communities, older adults, and people with disabilities by taking a multidimensional lens to participation in physical activity.

### Recommendations for future work

Findings from this rapid scoping review yielded valuable information about the scope of literature in the physical literacy landscape. Physical literacy as a body of research is expanding, with research actively being pursued and published. Current and recently published works from 2019 were included in the sample; however, given the literature search for this work was conducted in September of 2019, items published within the last quarter of 2019 were not included. Recent literature searches in December of 2019 have concluded articles of value to this rapid review are actively being published. Such is the case with a recently published article by Jefferies, Ungar, Aubertin, and Kriellaars [69] who demonstrated a link between resilience and physical literacy, indicating physical literacy may be linked to greater mental and social well-being.

This rapid review has identified key knowledge gaps. First, we recommend research dedicated to exploring the understudied domains of physical literacy - affective, cognitive, and behavioural - not only in children, but across the lifespan. While seven studies focused on physical literacy in children, only two articles in the original research sample studied physical literacy in adults [45, 46] and one studied physical literacy in older adults [49]. Research exploring physical literacy in adults and older adults is necessary to understand the potential role of physical literacy in the promotion of physical activity across the lifespan and the relationship of physical literacy to health and quality of life. Physical literacy should also be studied in special interest populations including those living with noncommunicable disease and people with physical disabilities. Second, there is a gap in the literature surrounding the practical applications and utilization of physical literacy in healthcare practice and research. Further research is required to better understand how health care providers can engage with the construct of physical literacy and where the construct can add value to practice. Finally, the relationship between physical activity, physical literacy, and health should be further elucidated in prospective studies to determine if physical literacy is a determinant of health, as has been proposed by previous authors [19].

### Limitations

In order to meet timelines and following other established rapid review approaches [70, 71], we had only one author complete title and abstract screening based on inclusion/exclusion criteria. Although all team members carefully reviewed all included articles during full text screening and any articles the reviewer was unsure about was discussed until consensus was reached, there is the potential this could have introduced bias in the review. As we were interested in exploring physical literacy as a holistic concept, our search term included the keyword ‘physical literacy’. A more comprehensive review may consider a search using keywords for each of the individual domains of physical literacy (e.g., motivation, affect) to more comprehensively determine the relationships between physical literacy domains and health using a framework analysis.

### Summary of key themes and findings

Based on tabulation coding and synthesis, the key themes and findings identified from the included literature are:

- Health care practitioners are not engaged with the construct of physical literacy in practice;
- Greater emphasis is placed on the physical domain of physical literacy, leaving the remaining domains (affective, cognitive, and behavioural) underrepresented and understudied;
- There is evidence to support a relationship between the physical domain of physical literacy and improved health outcomes, although the directionality of this relationship is unknown.

## Conclusions

This review advances understanding of the concept of physical literacy as it relates to health and the health care setting. Although there are emerging efforts to understand measurement of each individual domain of physical literacy, the utilization of this concept in the context of health is not well established or understood in the academic literature. The nature and direction of the relationship between physical literacy, physical activity, and health requires further exploration with consideration of the theoretical underpinnings of physical literacy as a holistic concept. This work is needed to clarify the role of physical literacy as a catalyst for promoting physical activity, decreasing the burden of disease, and increasing health and well-being.

## Supplementary information

**Additional file 1.** Protocol

**Additional file 2.** PRISMA-ScR checklist

**Additional file 3.** Search strategy

## Data Availability

All data generated or analyzed during this study are included in this publication article and its supplementary information files.

## Abbreviations

BMI: Body mass index
CAMSA: Canadian Agility and Movement Skills Assessment
CAPL: Canadian Assessment of Physical Literacy
CAPL-2: Canadian Assessment of Physical Literacy 2nd edition
PACER: Progressive Aerobic Cardiovascular Endurance Run
PLAYbasic: Physical Literacy Assessment for Youth basic
PLAYfun: Physical Literacy Assessment for Youth fun
PRISMA-P: Preferred Reporting Items for Systematic Reviews and Meta-Analysis Protocols
PRISMA-ScR: PRISMA extension for scoping reviews
SHAPE America: Society of Health and Physical Educators America
