# Supplementary material for: Understanding physical literacy in the context of health: a rapid scoping review
Source: BMC Public Health. 2020 Oct 19;20:1569. doi: 10.1186/s12889-020-09583-8 (PMC7570403; doi:10.1186/s12889-020-09583-8)
Supplement: Supplementary file 1 — Additional file 1. Protocol [file 12889_2020_9583_MOESM1_ESM.pdf]

UNIVERSITY OF NORTHERN BRITISH COLUMBIA

UNDERSTANDING THE CONSTRUCT OF PHYSICAL LITERACY IN THE CONTEXT OF HEALTH
Knowledge Synthesis Project Proposal

Prepared by:
UNBC HEALTH RESEARCH INSTITUTE
June 24, 2019

Working Title: Understanding physical literacy in the context of health: a rapid scoping review

UNBC Academic Lead:
Dr. Chelsea Pelletier, Assistant Professor, School of Health Sciences, UNBC

External Project Leads:
Gloria Fox, Physical Activity Lead, Northern Health
Dr. Anne Pousette, Executive Director, WINBC
Sabrina Dosanjh-Gantner, Regional Manager, Healthy Living, Chronic Disease Prevention & Public Health Practice, Northern Health

Background

The concept of physical literacy has gained momentum across British Columbia with the Physical Literacy for Communities (PL4C) Initiative spreading throughout BC communities. In the north, Prince George was the first community to participate in PL4C. The International Physical Literacy Association (IPLA) defines physical literacy as "the motivation, confidence, physical competence, knowledge and understanding to value and take responsibility for engagement in physical activities for life." (1). While the concept of physical literacy has evolved over time, the focus largely remains on fundamental movement skills related to sport and recreation participation. There is a lack of clarity when considering the construct of physical literacy in the context of health and as it relates to health care providers with most literature focused in the field of physical education. This work will help to develop an understanding of the use of physical literacy in the context of health and health provider education on the construct.

Purpose of the work:

The purpose of this work is to develop an understanding and definition of the physical literacy construct, as reflected in the context of health. Through a review of current literature, we aim to:

1) Define physical literacy as it relates to health across the lifespan by understanding how it has been conceptualized in health, as well as theorized and then implemented through interventions or practices;
2) Clarify the connection between physical literacy, physical activity levels, and improved health outcomes;
3) Clarify the role the health sector can play in promoting health via physical literacy enhanced activity.

The goal is to use this information in developing knowledge translation and educational products for healthcare providers to understand physical literacy and how it relates to physical activity and in turn improved health outcomes.

Proposed Methods

Project Team

This work will be conducted through a collaborative synthesis process with the assistance of the Knowledge Synthesis Centre at the University of Northern British Columbia Health Research Institute. The project team consists of a health sciences researcher and academic lead (CP), physical activity lead within the regional health authority (GF), physician and director of WINBC (AP), knowledge synthesis research associate (EK), health sciences librarian (TF), and research assistant (KC).

Literature review

This review will utilize a rapid review approach (2,3) to systematically identify and synthesize information on how physical literacy is defined and utilized in the context of health literature, identify physical literacy interventions within the health sector and related outcomes. Rapid reviews use a comprehensive, systematic approach to evidence review such as overview, realist, scoping, integrative, or traditional systematic methods but are completed in a condensed timeframe (4). The review will be structured as a rapid scoping review informed by the methodologically rigorous approach described by Arksey and O'Malley (5) and further developed by Levac et al (6) to systematically identify and synthesize information on how physical literacy is defined and utilized in the context of health literature, identify physical literacy interventions within the health sector, and related outcomes. This approach includes 1) identifying the

research question; 2) identifying literature (an iterative process); 3) selecting relevant studies; 4) extracting data; and 5) synthesizing, summarizing and reporting results.

Proposed Research Questions

1. How does current literature define physical literacy as it relates to health?
    a. What areas of health is the term physical literacy utilized and how are healthcare providers engaged with this construct?
2. Does existing literature describe a relationship between physical literacy, physical activity levels, and improved health outcomes, and if so, what are the key findings?

Identifying the literature

We will search the Medline, PubMed, Eric, Web of Science, and CINAHL databases at the University of Northern British Columbia for relevant health literature. Search strategies will be developed for individual databases with the assistance of a health research librarian using combinations of the terms physical literacy, physical and literate, health, perceived health, population health, physical fitness, wellbeing, wellness and quality of life. Keywords and subject headings were identified by reviewing the terms and search strategies used in previously published reviews on the topic and key articles identified by the research team. Reference lists of key papers identified by the project team and articles meeting inclusion criteria will be reviewed for additional papers that could be relevant and were not identified by the search. Articles will be selected for the review using inclusion and exclusion criteria identified with the project leads (including target population, study types and design, language, and jurisdictions).

Selecting Relevant Publications

The titles and abstracts of all identified articles will be uploaded into Distiller SR systematic review software (Evidence Partners, Ottawa, Canada) for screening and data extraction. In Distiller SR, all articles will be screened for inclusion or exclusion according to the predetermined eligibility (inclusion/exclusion) criteria. Screening will occur in two phases: 1) title and abstract and 2) full text. One reviewer (KC) will screen titles and abstracts to exclude clearly irrelevant articles before full texts are retrieved for articles considered relevant. One reviewer (KC) will screen full texts and the final list of articles reviewed by the study team to confirm articles for data extraction.

Table 1. Inclusion and exclusion criteria

Inclusion	Exclusion
(1) Articles which conceptualize or define physical literacy and are reported from a health context will be included. Studies must:<br>a. Have some mention of physical literacy or a physical literacy lens to the intervention<br>b. Reported from or in a health context including tools used in the past in a health setting or applied in the health sector<br>(2) All peer reviewed academic publications that are conceptual, theoretical, or original research articles will be included.	(1) Studies that do not conceptualize or define physical literacy.<br>(2) Case reports, conference abstracts, editorial and opinion pieces, book chapters, book reviews, and book synopses will be excluded.<br>(3) Non-English studies will be excluded.<br>(4) Articles where full text is not available through University of Northern British Columbia Library or Interlibrary Loans.

Extracting, Synthesizing and Reporting Data

Using Distiller SR characteristics of the included articles will be extracted including: author, title, year of publication, journal, country of origin, context, type of publication, study design and population. Details on how authors defined or conceptualized physical literacy, relationship to health, engagement of healthcare providers, key findings of interventions, and how they describe the relationship between physical literacy, physical activity levels, and improved health outcomes were also extracted. Articles will also be analyzed using thematic coding to identify themes across the body of literature, considering key words or phrases directly from text as well as implicit and explicit concepts for more

interpretive coding. Descriptive summary, graphical and tabular representations will be used to synthesize, summarize and report on the included publications, their characteristics, the concepts and outcomes assessed, and main findings.

Expected Outputs

Dr. Pelletier and the synthesis research team will submit the knowledge synthesis report in an electronic version to NH Team and Dr. Pousette. The evidence synthesized will be used to develop knowledge translation resources (i.e. fact sheet/pamphlet(s), presentation/online course outline) to be shared widely amongst health sector stakeholders wishing to support and advance the work of health enhancing physical activity and physical literacy for communities. The implementation of these resources will then be evaluated. We will present our findings locally through research institute events and webpage and the project team will also disseminate findings more broadly via academic and conference settings.

References

1. International Physical Literacy Association. IPLA About [Internet]. 2016. Available from: https://www.physical-literacy.org.uk/
2. Tricco AC, Langlois E V, Straus SE. Rapid Reviews To Strengthen Health Policy and Systems: a Practical Guide [Internet]. 2017. 119 p. Available from: http://apps.who.int/iris/bitstream/10665/258698/1/9789241512763-eng.pdf
3. Dobbins M. Rapid Review Guidebook: Steps for conducting a rapid review [Internet]. 2017. Available from: http://www.nccmt.ca/uploads/media/media/0001/01/a816af720e4d587e13da6bb307df8c907a5dff9a.pdf
4. Wilson MG, Lavis JN, Gauvin FP. Developing a rapid-response program for health system decision-makers in Canada: Findings from an issue brief and stakeholder dialogue. Syst Rev. 2015;4(1):1–11.
5. Arksey H, O'Malley L. Scoping studies: towards a methodological framework. Int J Soc Res Methodol. 2005;8:19–32.
6. Levac D, Colquhoun H, O'Brien KK. Scoping studies: Advancing the methodology. Implement Sci. 2010;5(1):1–9.
