# Supplementary material for: Understanding physical literacy in the context of health: a rapid scoping review
Source: BMC Public Health. 2020 Oct 19;20:1569. doi: 10.1186/s12889-020-09583-8 (PMC7570403; doi:10.1186/s12889-020-09583-8)
Supplement: Supplementary file 3 — Additional file 3. Search strategy [file 12889_2020_9583_MOESM3_ESM.pdf]

Medline OVID

Database(s): **Ovid MEDLINE(R) and Epub Ahead of Print, In-Process & Other Non-Indexed Citations and Daily** 1946 to August 13, 2019
Search Strategy:

Ovid®

Database(s): **Ovid MEDLINE(R) and Epub Ahead of Print, In-Process & Other Non-Indexed Citations and Daily** 1946 to August 30, 2019
Search Strategy:

#	Searches	Results
1	(physical* adj1 (literacy or literate)).ti,ab.	80
2	exp Attitude to Health/	396272
3	(health adj3 (perceived or perception or self-identified or self-satisfaction or attitude)).ti,ab.	15531
4	exp Public Health/	7447558
5	((public or population) adj1 health).ti,ab.	223823
6	exp Population Health/	38048
7	exp Physical Fitness/	27870
8	(physical adj1 fitness).ti,ab.	8842
9	(wellbeing or well-being or wellness).ti,ab.	89410
10	health.ti,ab.	1718558
11	"Quality of Life"/	180350
12	2 or 3 or 4 or 5 or 6 or 7 or 8 or 9 or 10 or 11	8379950
13	1 and 12	60
14	limit 13 to english language	60
15	from 14 keep 1-60	60
